# Diketopyrrolopyrrole-based semiconducting polymer nanoparticles for *in vivo* second near-infrared window imaging and image-guided tumor surgery†
†Electronic supplementary information (ESI) available. See DOI: 10.1039/c8sc00206a


**DOI:** 10.1039/c8sc00206a

**Published:** 2018-02-06

**Authors:** Kangquan Shou, Yufu Tang, Hao Chen, Si Chen, Lei Zhang, Ao Zhang, Quli Fan, Aixi Yu, Zhen Cheng

**Affiliations:** a Department of Orthopedics , Zhongnan Hospital of Wuhan University , Wuhan , Hubei 430071 , China . Email: yuaixi@whu.edu.cn; b Molecular Imaging Program at Stanford (MIPS) , Bio-X Program , Department of Radiology , Canary Center at Stanford for Cancer Early Detection , Stanford University , California 94305-5344 , USA . Email: zcheng@stanford.edu; c Key Laboratory for Organic Electronics and Information Displays , Institute of Advanced Materials (IAM) , Jiangsu National Synergetic Innovation Center for Advanced Materials (SICAM) , Nanjing University of Posts & Telecommunications , Nanjing 210023 , China . Email: iamqlfan@njupt.edu.cn; d CAS Key Laboratory of Receptor Research , Synthetic Organic & Medicinal Chemistry Laboratory (SOMCL) , Shanghai Institute of Materia Medica , Chinese Academy of Sciences , No. 555 Zuchong Road, Pudong New Area , Shanghai , P. R. China 201203

## Abstract

A diketopyrrolopyrrole-based semiconducting polymer nanoparticle (PDFT1032) has been developed as a NIR-II (near infrared window II, 1000–1700 nm) fluorescent probe. It shows high photostability, a favorable absorption peak at 809 nm, a large Stokes shift of 223 nm, outstanding biocompatibility and minimal *in vivo* toxicity. More importantly, the versatile use of PDFT1032 for several important biomedical applications in the NIR-II window has been demonstrated, including the NIR-II optical imaging of tumors on a subcutaneous osteosarcoma model, assessing the vascular embolization therapy of tumors, and NIR-II image-guided orthotopic tumor surgery and sentinel lymph node biopsy (SLNB) with high spatial and temporal resolution. Overall, excellent biocompatibility, favorable hydrophilicity, and desirable chemical and optical properties make the semiconducting polymer nanoparticle PDFT1032 a highly promising NIR-II imaging probe with the potential to be widely applicable in clinical imaging and the surgical treatment of malignancy.

## Abstract 2

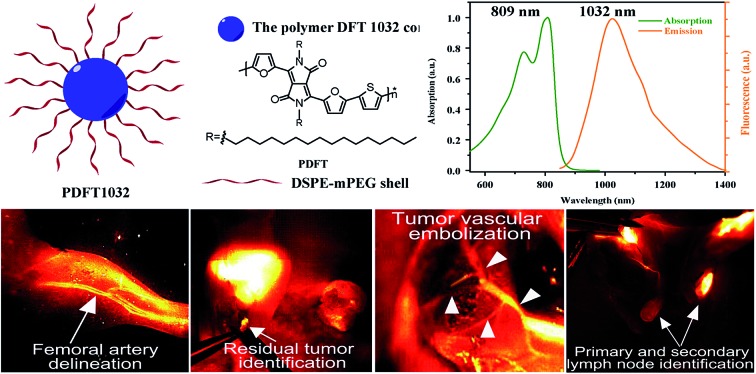
PDFT1032, a new semiconducting polymer possessing a favorable absorption peak (1032 nm) and outstanding biocompatibility, may be widely applicable in clinical imaging and the surgical treatment of malignancy.

## Introduction

*In vivo* fluorescence imaging in the second near-infrared window region (NIR-II, 1000–1700 nm) holds great promise for providing deeper tissue penetration and higher spatial resolution and signal-to-noise ratios than those obtained at the conventional near-infrared window I region (NIR-I, 650–900 nm), due to reduced tissue auto-fluorescence and photon scattering and low levels of long-wavelength photon absorption.^1^ To date, various ‘hard’ inorganic nanoparticles (NPs), such as single-walled carbon nanotubes (SWCNs), quantum dots (QDs) and rare-earth NPs, have been developed as NIR-II fluorescent agents for *in vivo* imaging.^2^ However, their potential long-term toxicity remains the primary roadblock for further biomedical applications. To achieve safety in preclinical models and potential clinical use, a handful of ‘soft’ organic materials have been reported for NIR-II fluorescence imaging which mainly rely on two types of organic small molecule, cyanine derivatives (IR1061) and benzobisthiadiazole derivatives.^3^,^4^ Unfortunately, most of the organic small molecules still suffer from limitations such as a low extinction coefficient and quantum yield, which may hinder the broad use of small molecule based NIR-II fluorescence imaging technology in biomedicine domains.^5^

Semiconducting polymer NPs represent a new class of fluorescent nanomaterial with excellent brightness that can be orders of magnitude higher than that of small-molecule fluorophores.^6^–^11^ Currently, due to their mostly emission wavelength (< 900 nm), semiconducting polymer NPs are particularly applied in NIR-I fluorescence imaging and exhibit good performance.^7^ However, in virtue of their limitations in molecular design and synthesis, the use of semiconducting polymer NPs for NIR-II imaging is still far behind that for NIR-I imaging. Furthermore, it is generally believed that the excitation wavelength at 808 nm can balance absorption and scattering to achieve a maximum tissue penetration depth greater than that of short wavelengths.^12^ The maximum permissible exposure for skin at a light intensity of 808 nm is also stronger than short wavelengths.^13^ Consequently, developing semiconducting polymer NPs with a strong absorption peak at 808 nm and excellent photostability and biocompatibility is highly desired for efficient *in vivo* NIR II imaging.^14^

**Fig. 1:**
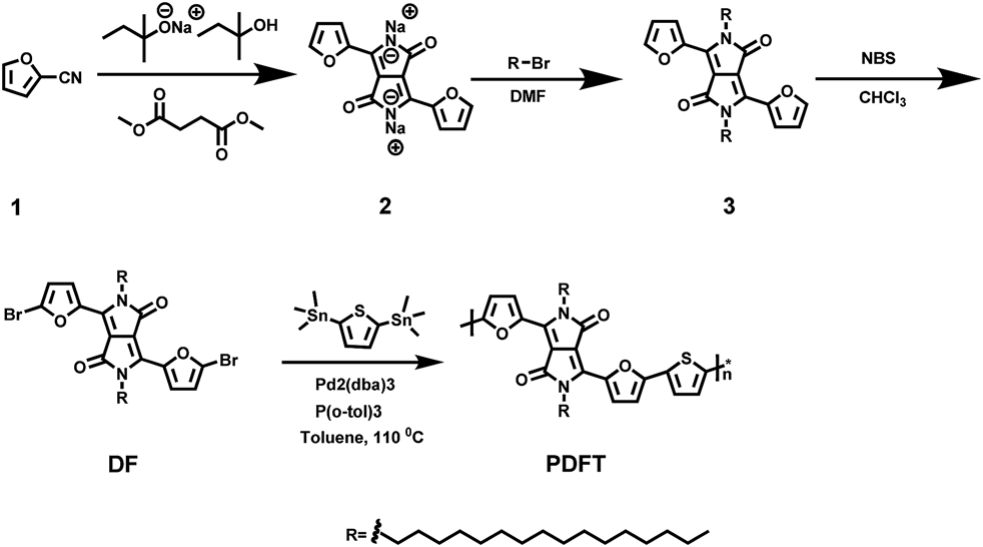
Synthetic route to PDFT. Detailed synthesis procedures can be found in the ESI.†

Diketopyrrolopyrrole (DPP), a planar electron acceptor, can easily couple with various electron donors to modify the bandgap and implement expected optical properties.^15^ Notably, DPP derivatives functionalized with electron-donor groups can exhibit red to NIR emission and a large Stokes shift, which are both quite suitable for biomedical imaging.^16^,^17^ Due to the excellent properties of maximum absorption between 600 and 800 nm and favorable light and thermal stability,^18^ DPP-based semiconducting polymers have been widely evaluated for photoacoustic (PA) imaging.^19^ Recently, DPP-based polymers have been applied to NIR-I fluorescence imaging, exhibiting good imaging performance including strong absorption in the NIR-I region, high biocompatibility, and excellent light and thermal stability.^16^ However, they are limited in permitting the visualization of microscopic biological structures within tissues in living objects because the emission wavelengths of all DPP-based polymers reported thus far are less than 900 nm,^17^,^18^,^20^ which is consequently difficult for the separation of excitation of fluorophores and image acquisition for real-time imaging. In this study, a semi-conducting polymer nanoparticle (named “PDFT1032”) was successfully designed and synthesized based on furan-containing diketopyrrolopyrrole polymers (PDFT) (Fig. 1), with an emission at the NIR-II window of 1032 nm. Then, we further evaluated and demonstrated its potential applications for efficient NIR-II *in vivo* imaging using multiple significant pathological models in living mice including NIR-II tumor imaging on a subcutaneous osteosarcoma model, assessing the vascular embolization therapy of tumors, and NIR-II image-guided orthotopic tumor surgery and real-time sentinel lymph node biopsy (SLNB) with high spatial and temporal resolution.

## Results and discussion

**Fig. 2:**
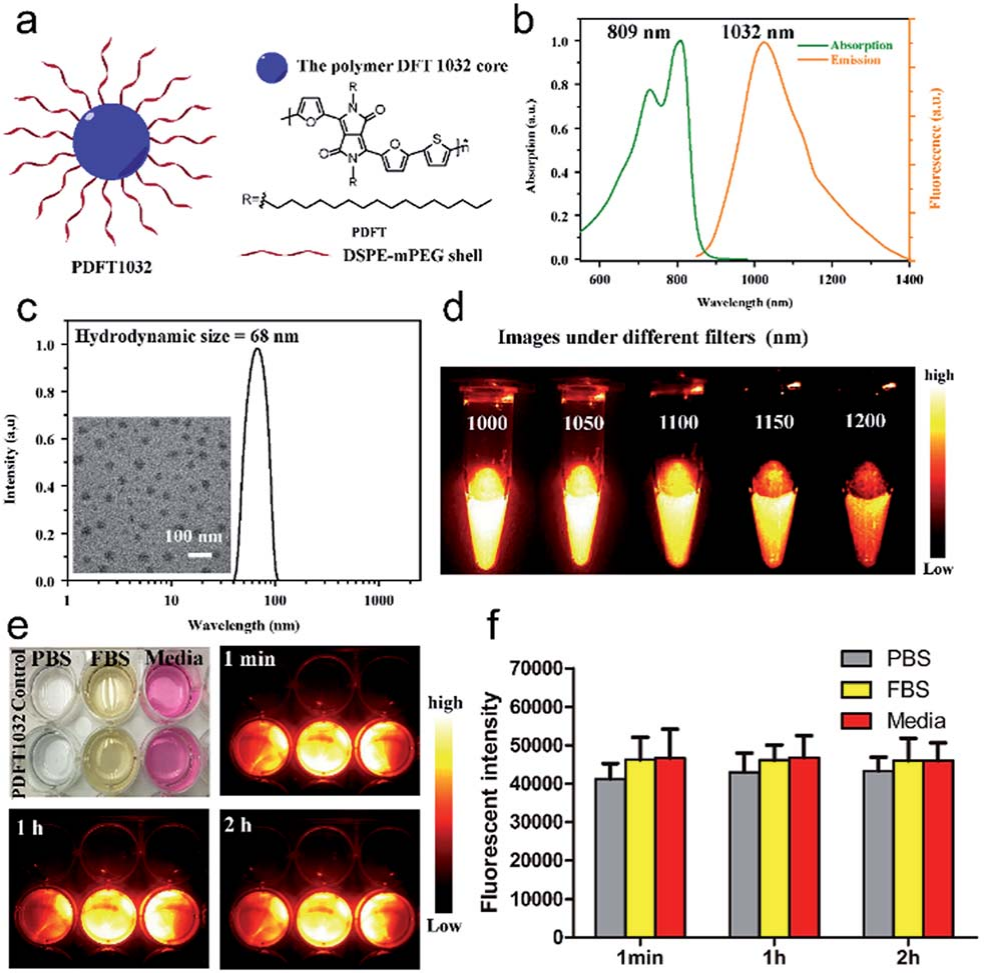
Characterization of PDFT1032. (a) Schematic drawing of a PDFT1032 nanoparticle composed of semiconducting polymer DFT and a hydrophilic DSPE-mPEG shell. (b) Absorbance and fluorescence spectrum of PDFT1032 showing an absorption peak at 809 nm and a fluorescence peak at 1032 nm with an 808 nm excitation laser. (c) A DLS spectrum and TEM image (inset) of PDFT1032, showing a hydrodynamic size of 68 nm. (d) NIR-II signals of PDFT1032 with sequential long-pass filters (1000–1200 nm), indicating that the fluorescence signals of PDFT1032 were distinct at the 1000 and 1050 nm filters, and even above 1200 nm. A photostability test of PDFT1032 using NIR-II fluorescence imaging (e) and quantified fluorescence intensity (f) for PDFT1032 in PBS, FBS and DMEM (cell culture media) with 808 nm excitation laser radiation for up to 2 h. The photobleaching of PDFT1032 in PBS, FBS and DMEM culture media was almost negligible.

In our study, PDFT1032 exhibits an excellent maximum absorption wavelength at 809 nm (Fig. 2). For the first time, we designed and prepared the novel DPP-based semiconducting polymer (PDFT) with a low bandgap through donor–acceptor (D–A) alternating copolymerization. The PDFT used thiophene (T) as the donor and 2,5-dihexadecyl-3,6-di(furan-2-yl)pyrrolo[3,4-*c*]pyrrole-1,4(2*H*,5*H*)-dione (DPP-2F) as the acceptor. Hydrophobic PDFTs were then encapsulated with the amphiphilic PEGylated phospholipid (DSPE-mPEG, *M*_w_ = 5 kDa) to provide excellent water-soluble and biocompatible PDFT1032s. The PDFT1032s showed high monodispersity and homogeneity with a particle size of 68 nm in PBS (Fig. 2c and S5 in ESI†). Impressively, the particle size of PDFT1032 was almost unchanged in PBS at 30 days, indicating good stability in PBS (Fig. S6 in ESI†).

Furthermore, PDFT1032 showed a unique absorption peak at 809 nm (Fig. 2b). As expected, the fluorescence emission spectrum of PDFT1032 was in the NIR-II region (a main emission peak at 1032 nm in PBS), exhibiting a large Stokes shift of 223 nm (Fig. 2b). The NIR-II fluorescence emission signals of PDFT1032 under different long pass (LP) filters (1000–1200 nm) showed that the fluorescence signals of PDFT1032 were apparent at the 1000 and 1100 nm filters, and even at 1200 nm (Fig. 2d and S6 in ESI†). In addition, the decay of fluorescence intensity of PDFT1032 in PBS, FBS and DMEM culturing media was almost negligible under continuous 808 nm light at a power density of 140 mW cm^–2^, even over 2 h (Fig. 2e and f). Previously, a semiconducting polymer NP named ‘pDA-PEG’ has been used for NIR-II imaging.^1^ However, serious concerns still remain regarding the low photostability (the reported fluorescence intensity of pDA-PEG declines by 20% under an excitation of 808 nm for 1 h) and unfavorably short absorption wavelength (an absorption peak at 654 nm) of pDA-PEG.^1^ In comparison, PDFT1032 showed maximum absorption at 809 nm, which is much more favorable for *in vivo* imaging owing to deeper tissue penetration depth and stronger maximum permissible exposure for skin at a light intensity of 800 nm. Rao *et al.*^21^ previously reported a NIR-I fluorescent nanoprobe, synthesised by encapsulating NIR-I dye into the conjugated polymer (MEH-PPV), and the *in vivo* biodistribution and bioimaging of the nanoprobe in living mice were demonstrated. However, the photostability of the MEH-PPV probe is poor. On the contrary, the PDFT1032 probe reported here has very high photostability. Overall, our results shown here suggest that PDFT1032 is obviously well suited for bioimaging in the NIR-II window on account of its high photostability and favorable absorption.

To further use PDFT1032 as a NIR-II imaging agent *in vivo*, it is necessary to evaluate its potential toxicity. *In vitro* cytotoxicity was first studied by standard MTT analysis. No apparent cytotoxicity of PDFT1032 was observed even in concentrations up to 150 μg mL^–1^, indicating its low cytotoxicity (Fig. S7a†). The IC_50_ of PDFT1032 was further determined to be 347 μg mL^–1^, much higher than that of pDA-PEG (30 μg mL^–1^). To evaluate the long term effects of PDFT1032 on animals, C57BL/6 mice were intravenously injected with PDFT1032 in doses of 0.5 and 1 mg kg^–1^, which are close to the normal dosage and overloaded dosage used in *in vivo* experiments, and PBS solution was used as a control. Almost no differences in body weights between the control and experimental groups each day for 30 days was observed (Fig. S7b†). In addition, H&E staining of major organs, including the heart, liver, spleen, lung, and kidney, demonstrated no obvious hydropic damage or necrotic lesions at 21 days after intravenous injection of PDFT1032, indicating its potentially good long term biocompatibility *in vivo* (Fig. S7c†). Thus, our findings clearly demonstrate that PDFT1032 exhibited much more favorable biocompatibility compared with previously reported pDA-PEG, indicating that PDFT1032 is more applicable for clinical translation as an organic polymer based NIR-II nanoprobe.

To understand the *in vivo* clearance and biodistribution of PDFT1032, the nanoprobe (200 μL, 50 μg mL^–1^) was injected into C57BL/6 mice (*n* = 3) through the tail vein. An *Ex vivo* biodistribution study was performed by NIR-II imaging of the vital organs, including the heart, liver, spleen, lung, kidney, brain and skin, 24 h post-injection of PDFT1032. The results revealed low uptakes in the heart, brain and skin (Fig. S8a and b in ESI†). All these data implied that PDFT1032 is suitable for *in vivo* NIR-II imaging. Additionally, NIR-II imaging of PDFT1032 on mice hind limb vessels (femoral arteries) was also investigated. It was found that the hind limb vessel was distinguishingly observed under NIR-II imaging and the fluorescence signal of PDFT1032 was maintained for several minutes (Fig. S9 in ESI†), indicating that PDFT1032 is appropriate for vascular imaging during surgery.

**Fig. 3:**
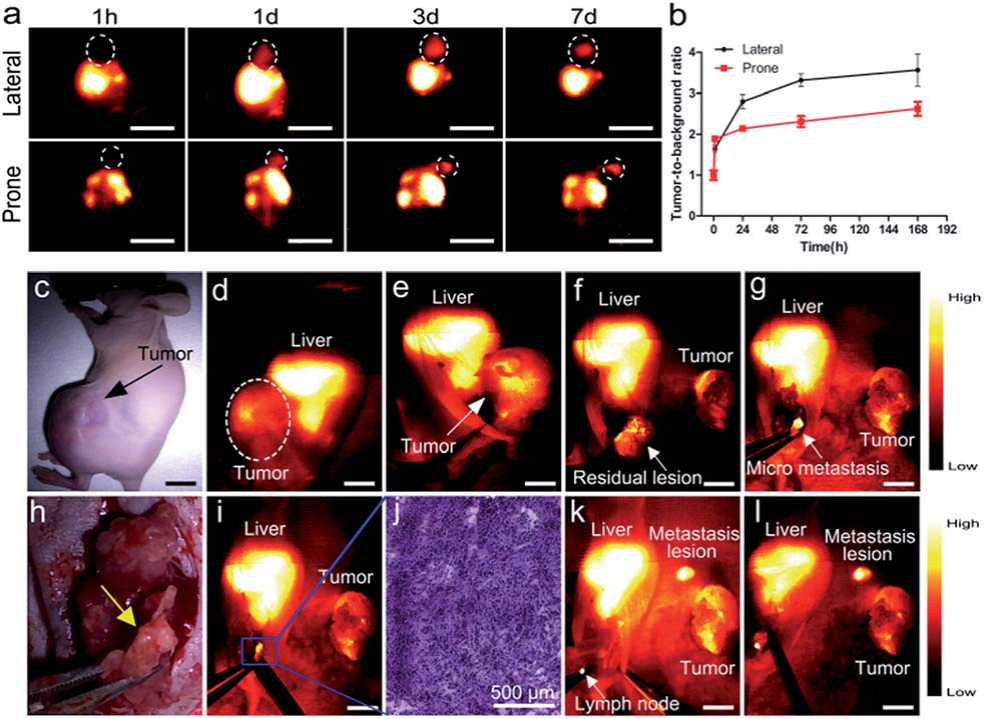
NIR-II imaging and image-guided surgery on mice with osteosarcoma using PDFT1032. (a) The images from both the lateral and prone position in mice (*n* = 3) demonstrated clear visualization of the subcutaneous tumor up to 7 days. (b) High tumor-to-background ratio of the lateral position (TBR, up to 3.5 ± 0.39) and the prone position (up to 2.6 ± 0.16) due to the excellent EPR effect of PDFT1032. (c) Bright field photograph of a nude mouse with an orthotopic osteosarcoma. The whole tumor was visualized using NIR-II imaging with excellent contrast ((d) and (e)). The white dashed circle contours the location of the tumor. (f) The tumor body was resected. The NIR-II signal still remained around the knee joint, indicating that there were adjacent tumor metastases located at the metaphysis of the tibia. The white arrow indicates the residual lesion. (g) The micro-metastasis of the tumor is identified using NIR-II imaging, and (h) the bright field photograph of the surgical scope. (i) The micro-metastasis was then completely resected using NIR-II imaging, which was further confirmed by the histological analysis (j). Finally, the lymph node was also visualized and resected with the help of PDFT1032 (k) and (l). Scale bar: 1.5 cm.

To examine the capability of PDFT1032 for NIR-II tumor imaging and image-guided surgery *in vivo*, the nanoprobe (200 μL, 50 μg mL^–1^) was injected into mice (*n* = 3) with a subcutaneous osteosarcoma at the right shoulder, through the tail vein. The NIR-II images of both the lateral and prone positions at different time-points demonstrated the clear visualization of the tumor for up to 3 days with a high tumor-to-background ratio (TBR, up to 3.4 ± 0.16 for the lateral position and 2.4 ± 0.15 for the prone position), which is attributed to the accumulation of the nanoprobe through the enhanced permeability and retention (EPR) effect (Fig. 3a and b). *Ex vivo* fluorescence images also demonstrated the favorable contrast between the tumor and the skin due to the low fluorescence signal of skin (Fig. S10, ESI†). The dynamic imaging and biodistribution studies clearly show that the nanoprobe can be cleared by both the kidneys and the hepatobiliary systems. High tumor uptakes and retention were also observed. Overall, PDFT1032 exhibited a desirable NIR-II tumor imaging capability owing to good TBR (∼3.5), which also suggests it is suitable for NIR-II image-guided surgery.

To confirm the ability of PDFT1032 in NIR-II image-guided tumor surgery, the mice with an orthotopic osteosarcoma (*n* = 3) were visualized using NIR-II imaging with excellent contrast (Fig. 3c and d). Thanks to the micron-scale spatial resolution and high temporal resolution (>25 frames per second), the NIR-II window imaging showed high sensitivity for the delineating orthotopic tumor. After the imaging-guided tumor resection was completed, there were still NIR-II signals remaining around the knee joint, which were otherwise not visible to the naked eye of the surgeon (Fig. 3f and h). Therefore, the resection bed was surveyed to eliminate all of the residual lesions. Interestingly, the lesions, including adjacent orthotopic tumor micro-metastasis (Fig. 3g–i) (which was further confirmed by histological analysis, Fig. 3j) as well as the lymph node (Fig. 3k–l), were then identified and completely resected under the guidance of our NIR-II imaging technique. Impressively, the NIR-II imaging PDFT1032 displayed a high sensitivity towards visualizing orthotopic tumors and their micro-metastasis (satellite lesions), thereby providing us with the ability to resect residual lesions and micro-metastasis accurately, thus eliminating relapses as much as possible. Interestingly, a series of nanoparticles using the DPP-based semiconducting polymer were synthesized and used for tumor photoacoustic imaging after an intravenous injection into mice.^19^ However, it is a challenge to use photoacoustic imaging for tumor resection due to its low temporal resolution,^22^ because the long interval time between the action of the surgeon and the feedback of the signal would evidently intervene or distract the intraoperative judgement and the decision of the surgeon. Hence, the excellent temporal resolution of NIR-II imaging renders PDFT1032 a reliable and promising probe for real-time image-guided surgery on patients.

**Fig. 4:**
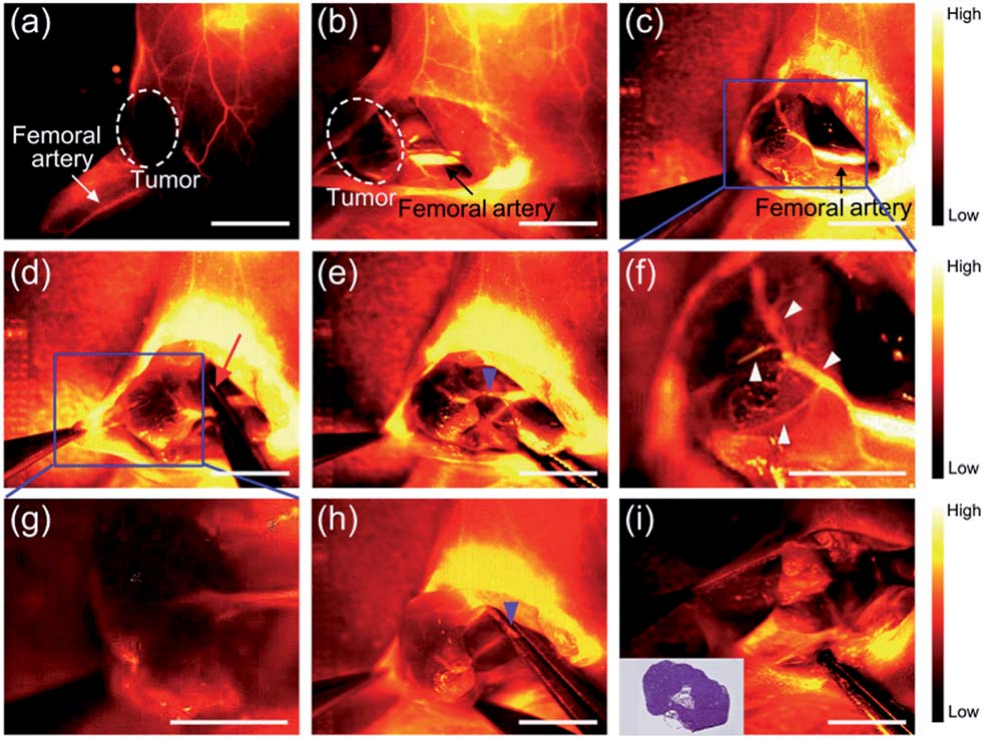
NIR-II imaging for the assessment of the vascular embolization therapy of osteosarcoma with PDFT1032. (a) The vascular mapping and the hemodynamic status of the tumor and the femoral artery were determined. The white dashed circle contours the location of the tumor. ((b) and (c)) The branch of the femoral artery that supports the tumor and the vascular network of the tumor (exhibited as a claw shape) were clearly identified. (d) A vessel clamp was used to block the blood flow (red arrow) and the signal of the vascular network vanished. (e) After 5 minutes, the clamp was removed and the blood flow of the tumor was still devoid because a temporary thrombus was formed (blue arrowhead). (f) Magnification of (c). The vascular network of the tumor was clearly identified (white arrowheads). (g) Magnification of (d). (h) The major artery was surgically incised (blue arrowhead). (i) NIR-II imaging exhibited the absence of the residual tumor fluorescence and normal circulation (femoral artery) was successfully maintained. Inset is the histological analysis of the osteosarcoma. Scale bar: 8 mm.

Furthermore, for patients who show a poor response to chemotherapy or have recurrent or unresectable tumors, vascular embolization may be an alternative therapy to reduce pain in a short period of time.^23^,^24^ To evaluate the ability of PDFT1032 as a NIR-II probe for imaging the major blood vessels of tumors in order to implement embolotherapy, PDFT1032 (200 μL, 50 μg mL^–1^) was injected intravenously into mice (*n* = 3) with a tumor located at the proximal femur. Five minutes later, the vascular mapping and the hemodynamic status of the tumor, as well as the femoral artery, were well-determined using the PDFT1032 nanoprobe (Fig. 4a and b). It is of note that the branch of the femoral artery that supports the tumor and the vascular network of the tumor (exhibited as a claw shape) were clearly identified (Fig. 4c and f). Next, the procedure mimicking vascular embolization was performed by a skilled surgeon according to the NIR-II imaging results with a 1000 nm LP filter and 200 ms exposure time. To accomplish the occlusion of the major blood supply and the collateral circulation, a vessel clamp was utilized to block the blood flow and hence the signal of the vascular network vanished successfully (Fig. 4d and g). After 5 min, the clamp was removed and the blood flow of the tumor was still devoid because a temporary thrombus was formed (Fig. 4e). Next, the major artery was surgically incised (Fig. 4h) and the tumor was resected, while normal circulation (femoral artery) was successfully maintained with the help of the NIR-II image-guided surgery and further confirmed by pathological examination (Fig. 4i).

Sentinel lymph node biopsy (SLNB) mapping has been widely applied in clinical practice for predicting the metastatic spread of tumors.^25^,^26^ For visualizing axillary lymph nodes, PDFT1032 (50 μL, 50 μg mL^–1^) was injected intradermally into the dorsal skin of the forepaws of four to six-week-old nude mice (*n* = 3). With the help of NIR-II imaging, the axillary lymph node was clearly identified within a short time (Fig. S11a–c in ESI†), dissected from the ambient tissue (Fig. S11d and e in ESI†), and then confirmed by histological analysis (Fig. S11m in ESI†). To further test the feasibility of using PDFT1032 as a fluorescent tracer on melanoma B16F10-bearing C57BL/6 mice (*n* = 3), PDFT1032 (50 μL, 50 μg mL^–1^) was injected into the forepaw near the melanoma located at the right shoulder. Ten minutes later, the axillary lymph node was notably identified (Fig. S11g in ESI†). Interestingly, the afferent lymphatic vessels for both the sentinel lymph node and the secondary lymph node gradually became visible and distinguishable at 7 min post-injection (Fig. S11h in ESI†), demonstrating the successful visualization of SLNB using PDFT1032 (Fig. S11i and j in ESI†). During the surgical procedure, the sentinel lymph nodes were distinctly identified and distinguished from the visual field because of the favorable signal-to-background ratio (SBR) generated by the NIR-II PDFT1032 probe (Fig. S11k and l in ESI†). Hence, our findings demonstrated that the NIR-II probe PDFT1032 exhibited excellent imaging quality for the identification of SLNs both on normal mice and tumor-bearing mice.

**Fig. 5:**
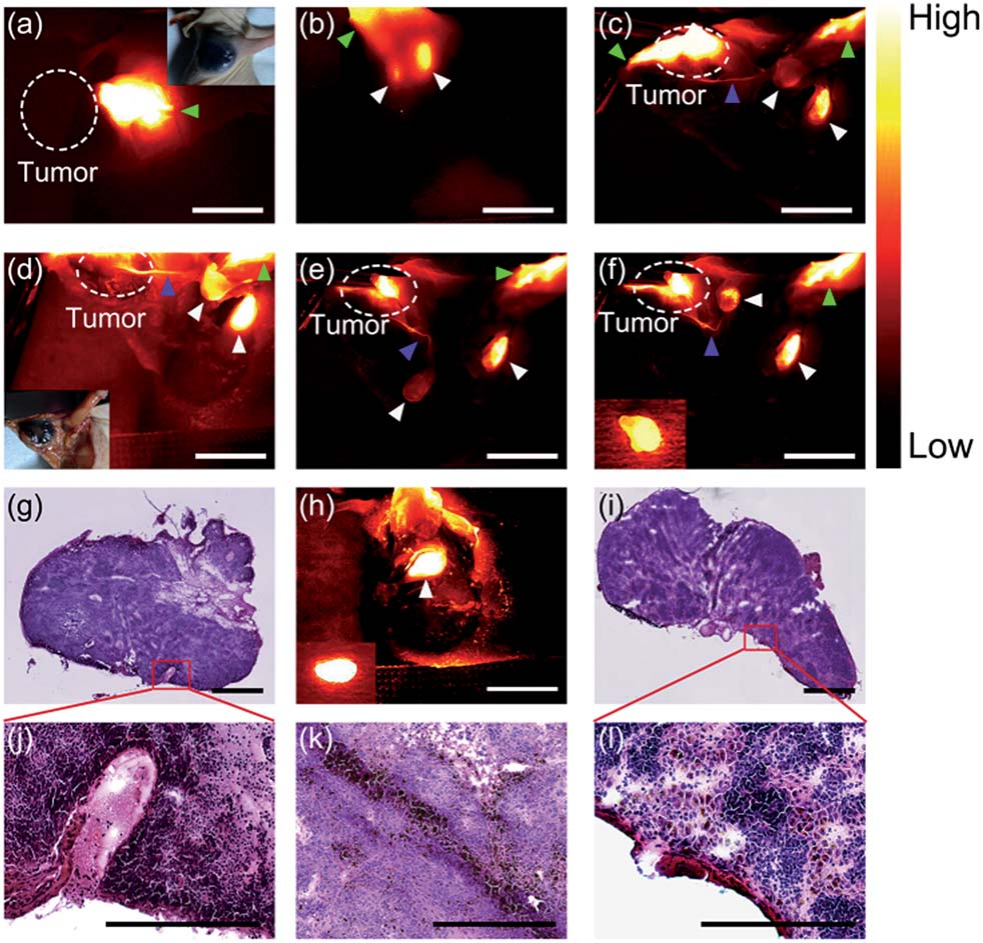
NIR-II image-guided sentinel lymph node biopsy on one of the mice with melanoma. (a) PDFT1032 (green arrowhead) was injected at the margin of the B16F10 melanoma (white dashed circle). (b) Ten minutes later, the two axillary lymph nodes were clearly identified (white arrowhead). (c)–(e) The efferent lymphatic vessel connecting the tumor with the node (blue arrowhead) was clearly identified. The SLN and the secondary lymph node were distinctly identified. (f) The SLN was resected (inserted image). After a positive result for the SLN was confirmed ((g) and (j)), the secondary lymph node was subsequently resected (h). Finally, histological analysis also confirmed the metastasis of melanomas ((i) and (l)). (k) The histological result of a resected melanoma, colored black. For the fluorescence images, scale bar: 8 mm, for the histological analysis, scale bar: 75 μm.

To mimic the standard sentinel lymph node biopsy procedure used in clinical practice, nude mice (*n* = 5) with a melanoma B16F10 on the right shoulder were used. To trace real lymphatic flow and drainage, PDFT1032 (50 μL, 50 μg mL^–1^) was injected intradermally at the margin of the tumor and thus its lymphatic route would be shared with the tumor (Fig. 5a). Ten minutes later, two axillary lymph nodes were clearly identified with the skin intact (Fig. 5b). With the help of NIR-II imaging, the SLNs were visualized and resected. Histological analysis of the SLNs was performed to examine whether tumor metastasis in SLNs existed or not. If the histological finding was positive, the secondary lymph nodes (axillary) were resected sequentially using NIR-II imaging and then embedded by an OCT compound to perform the histological analysis to confirm tumor metastasis. If the histological finding of the SLN was negative, the survey was completed. Notably, the detection rate and the false-negative rate of SLNs were calculated based on the successful determination of lymph nodes and the histological analysis. After the soft tissue was dissected and separated, the efferent lymphatic vessel connected to the tumor and lymph node was clearly identified (Fig. 5c and d), ascertaining that the first lymph node was the SLN. Consequently, we dissected the SLN from its soft tissue bed and the luminous NIR-II signal aided us to discriminate the SLN clearly at different positions (Fig. 5e and f). Then, the SLN was resected and the histological analysis which mimics the rapid pathological examination during tumor surgery on patients was performed. After a positive result was confirmed by an experienced pathologist (Fig. 5g and j), the secondary lymph node was subsequently resected according to the NIR-II imaging guidance (Fig. 5h). Finally, the histological analysis also confirmed the metastasis of melanoma in the second sentinel lymph node (Fig. 5i and l), proving the necessity of the continued survey. Moreover, the SLNs of all five mice were detected successfully (success rates of detection = 100%), while only one mice was diagnosed with no metastasis existing in SLN but the secondary LN was further confirmed positive with metastasis (false-negative rate = 20%). Currently, indocyanine green (ICG) has been successfully applied on patients for lymphatic system assessments and SLNB, but it has been recognized that the relatively poor long term *in vivo* stability of ICG may hinder its further applications.^27^,^28^ Additionally, there is still a high false-negative rate (46%) reported for the use of ICG for SLNB.^29^ On the contrary, our preliminary results in animal models showed a 100% detection rate for the SLN and 20% for the false-negative rate attributed to the outstanding optical properties of PDFT1032, highlighting that NIR-II image-guided SLNB with PDFT1032 may serve as a more favorable strategy with a higher detection value than that of ICG. Notably, the afferent lymphatic vessels were also clearly identified during the process of SLNB, indicating that several pathological alterations of lymphatic drainage routes generated by tumors may also be dynamically monitored using the NIR-II nanoprobe. This finding is important, and the therapy used in this study mimics more closely the real procedure used in clinical practice, especially for the intraoperative frozen section examination, compared with our previous report using a small organic molecule based NIR-II probe for SLNB.^30^

## Conclusions

In summary, this study unambiguously shows that a new semiconducting polymer NP (PDFT1032) possesses various excellent properties for *in vivo* NIR II imaging, including high photostability, a favorable absorption peak, a large Stokes shift of 223 nm, outstanding biocompatibility, and minimal *in vivo* toxicity. This probe is able to achieve several meaningful biomedical real-time imaging applications including tumor diagnosis and SLN mapping. More importantly, NIR-II imaging using PDFT1032 is capable of aiding us to accomplish accurate image-guided tumor surgery, vascular embolization therapy and image-guided SLNB with high spatial and temporal resolution. Excellent biocompatibility, favorable hydrophilicity, and desirable chemical and optical properties render NIR-II PDFT1032 a highly promising probe with the potential to be widely applicable in clinical imaging and the surgical treatment of malignancy.

## Author contributions

Z. C., Q. F. and A. Y. conceived and designed the experiments. K. S., Y. T., H. C., S. C., and L. Z. performed all experiments. Y. T. contributed to the design and synthesis of the semi-conducting polymer. K. S. and H. C. contributed to the preparation of the organic nanoparticles, the optical characterization of the nanoparticles and the animal experimentation. K. S., Z. C., Y. T., A. Z., and H. C. analyzed the data and wrote the manuscript. All authors discussed the results and commented on the manuscript.

## Conflicts of interest

There are no conflicts to declare.

## Supplementary Material

Supplementary information
