# Supplementary material for: Diketopyrrolopyrrole-based semiconducting polymer nanoparticles for in vivo second near-infrared window imaging and image-guided tumor surgery
Source: Chem Sci. 2018 Feb 6;9(12):3105–10. doi: 10.1039/c8sc00206a (PMC5914543; doi:10.1039/c8sc00206a)
Supplement: Supplementary file 1 [file SC-009-C8SC00206A-s001.pdf]

Electronic Supplementary Material (ESI) for Chemical Science.
This journal is © The Royal Society of Chemistry 2018

Supporting Information

Diketopyrrolopyrrole-Based Semiconducting Polymer Nanoparticles for In Vivo Second Near-Infrared Window Imaging and Imaging-Guided Tumor Surgery

Kangquan Shou[a,b†] , Yufu Tang[c†], Hao Chen[b†], Si Chen[b], Lei Zhang[b], Quli Fan*[c], Aixi Yu*[a], Zhen Cheng*[b]

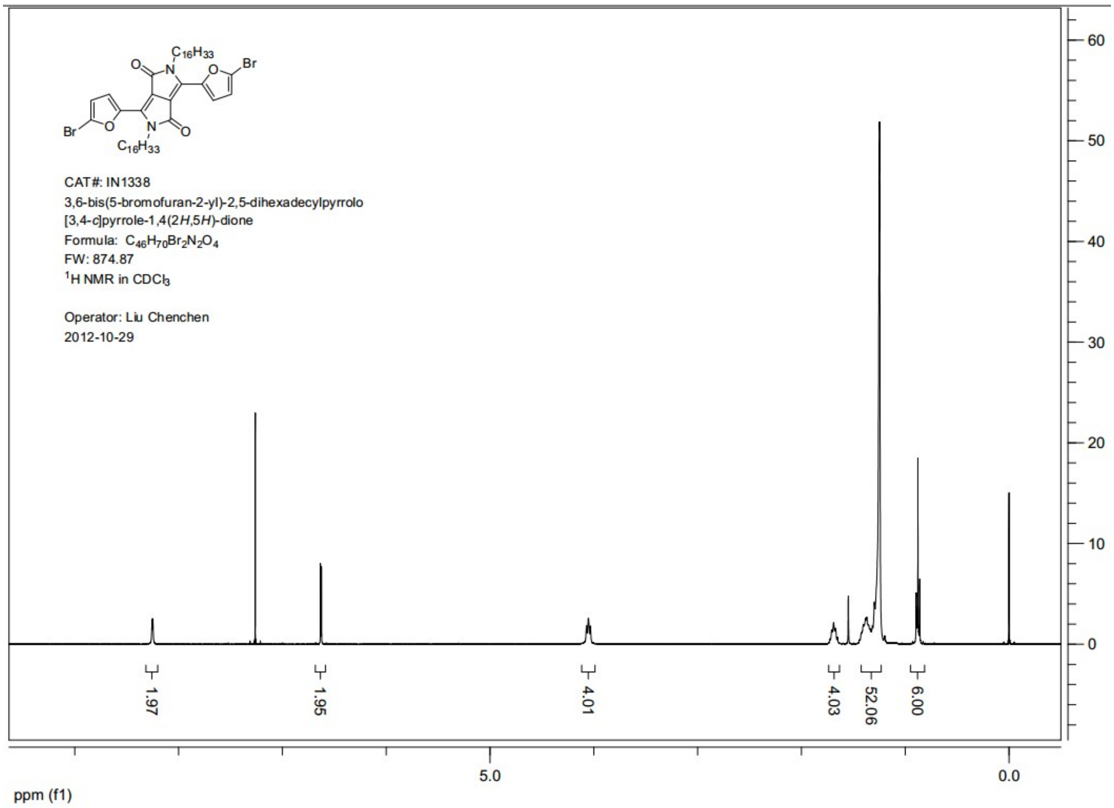

Figure S1. ^1H NMR Spectrum of the DF in $CDCl_3$.

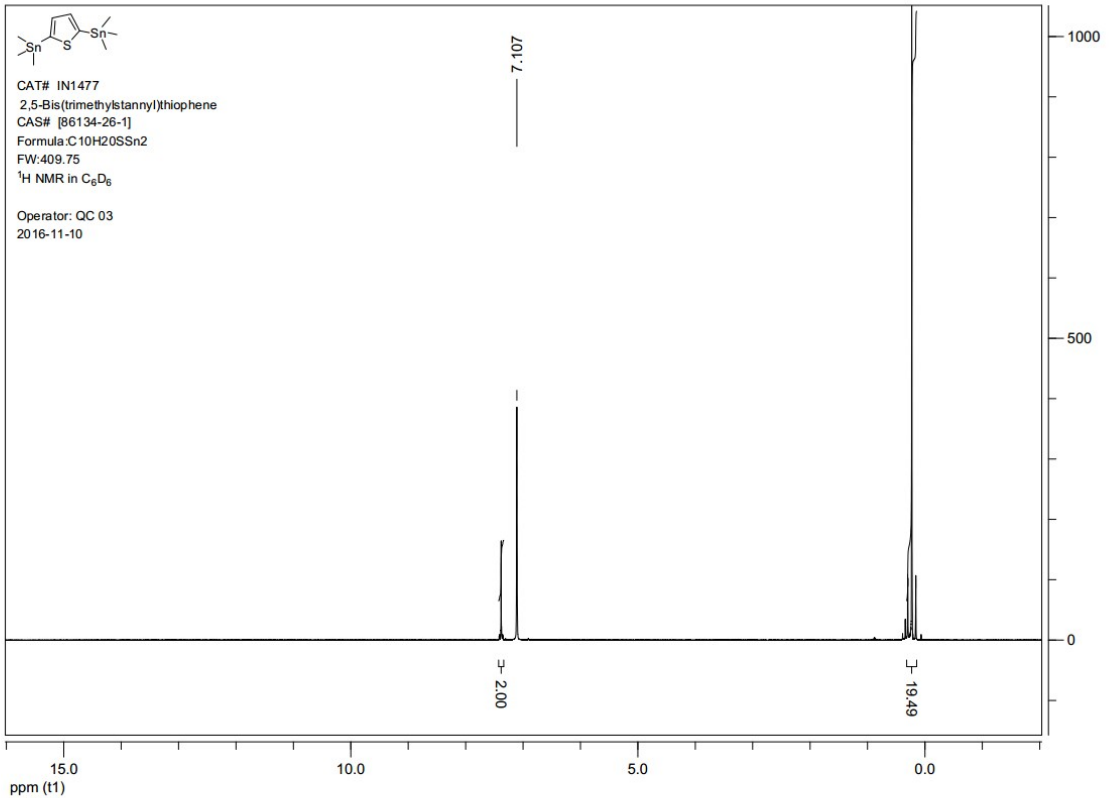

Figure S2. ^{1}H NMR Spectrum of the T in C_6D_6.

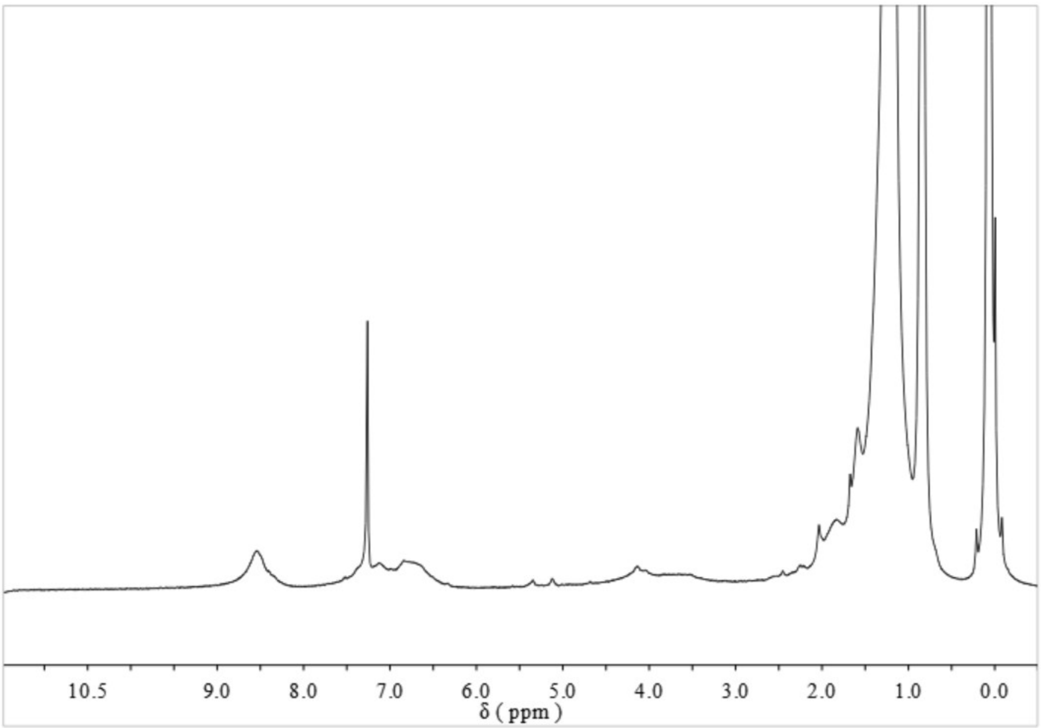

Figure S3. ^{1}H NMR Spectrum of the PDFT Polymer in $CDCl_3$. NMR spectra were recorded on a Bruker Ultra Shield Plus 400 MHz spectrometer (1 H, 400 MHz) using tetramethylsilane (TMS) as the internal standard. PDFT shows significant broadening of the aromatic peaks, alongside a minor shift upfield to 8.43 and 6.66 ppm. PDFT polymers display alkane peaks at 1.35 ppm.

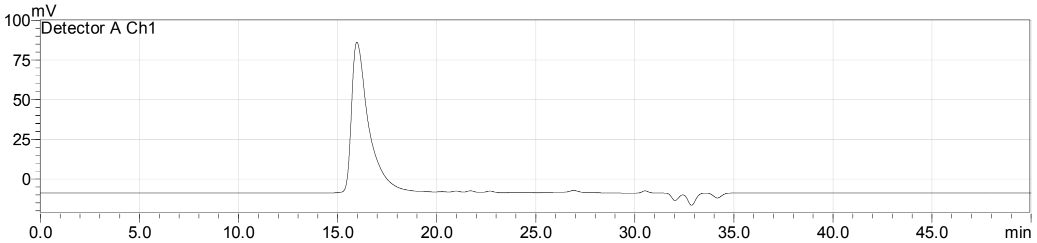

Figure S4.Gel Permeation Chromatography (GPC) Spectrum of PDFT. Mn (number-average molecular weight): 52 kDa; Mw (weight-average molecular weight): 80 kDa; PDI (polydispersity index):1.86.

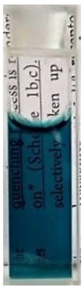

Figure S5. Picture of PDFT1032 (50 μg/ mL) in water.   Hydrodynamic size= 68 nm.

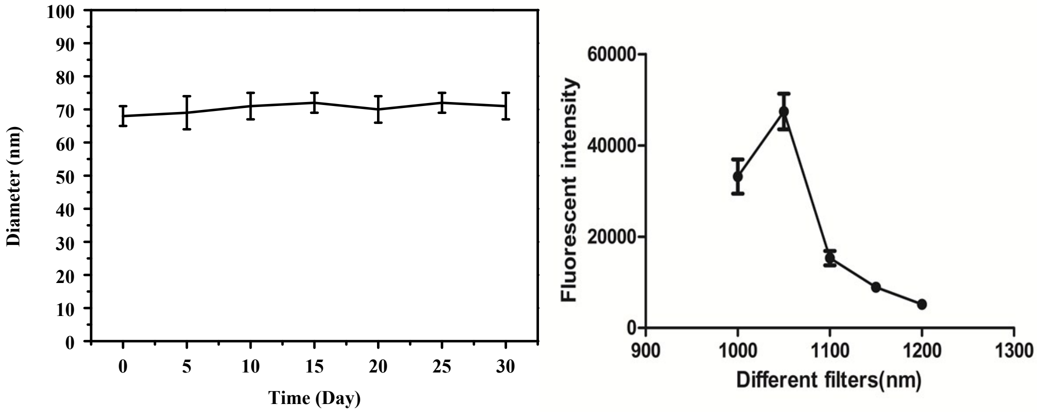

Figure S6. **(a)** Hydrodynamic diameters of the PDFT1032 in PBS for different time periods. **(b)** Quantification of the fluorescent intensity of PDFT1032 under different filters.

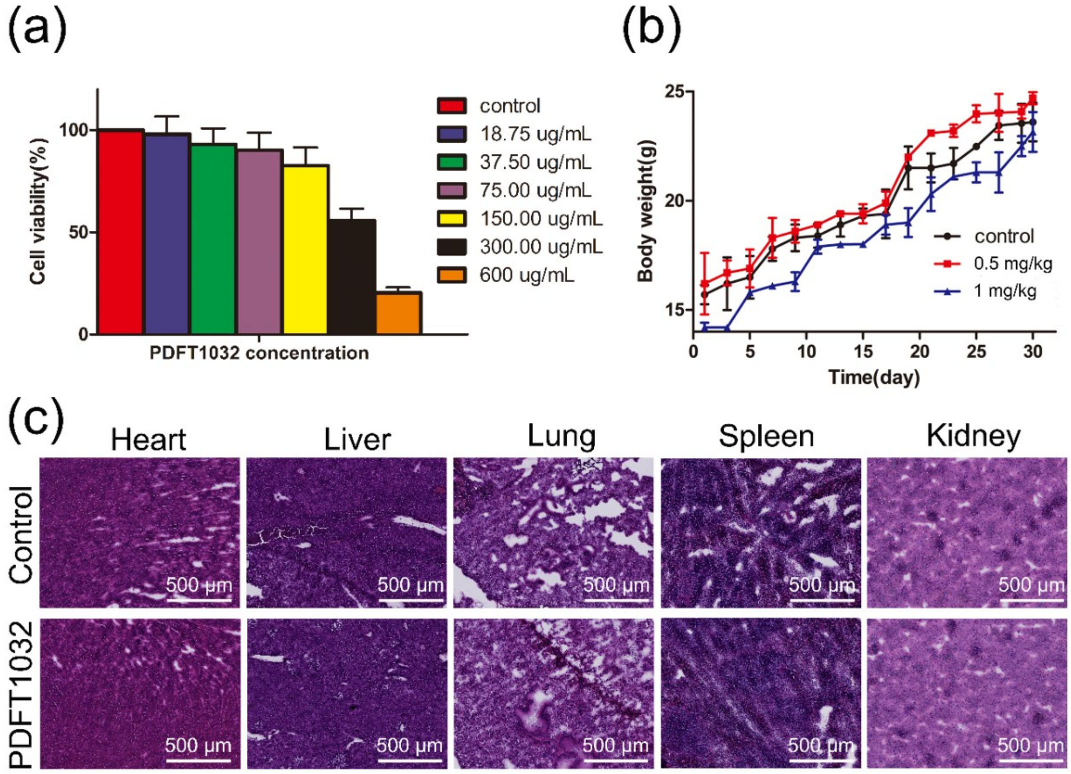

Figure S7. Biocompatibility of PDFT1032. (a) MTT assay of PDFT1032, indicating that there was no observed toxicity to NIH3T3 cells with different concentrations of PDFT1032 tested. (b) Body weight of the mice (n=3) at different days post treatment with PDFT1032 at the dose of 0.5 and 1 mg/kg. (c) Representative H&E stained images of major organs including heart, liver, spleen, lung and kidney collected from the untreated mice and PDFT1032 injected mice at 21 days post-injection of the nanoprobe (0.5mg/kg). No obvious organ damage or lesion was observed for PDFT1032 treated mice. Scale bar: 500 μm.

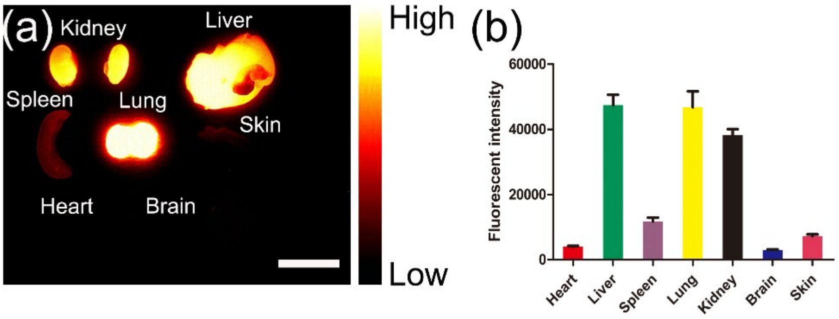

Figure S8. ***Ex vivo*** **biodistribution of PDFT1032. (a)** *Ex vivo* biodistribution proved the minimal auto-fluorescence of skin. The ex vivo fluorescent signal of different organs were shown as **(b)**. Scale bar: 1.5 cm.

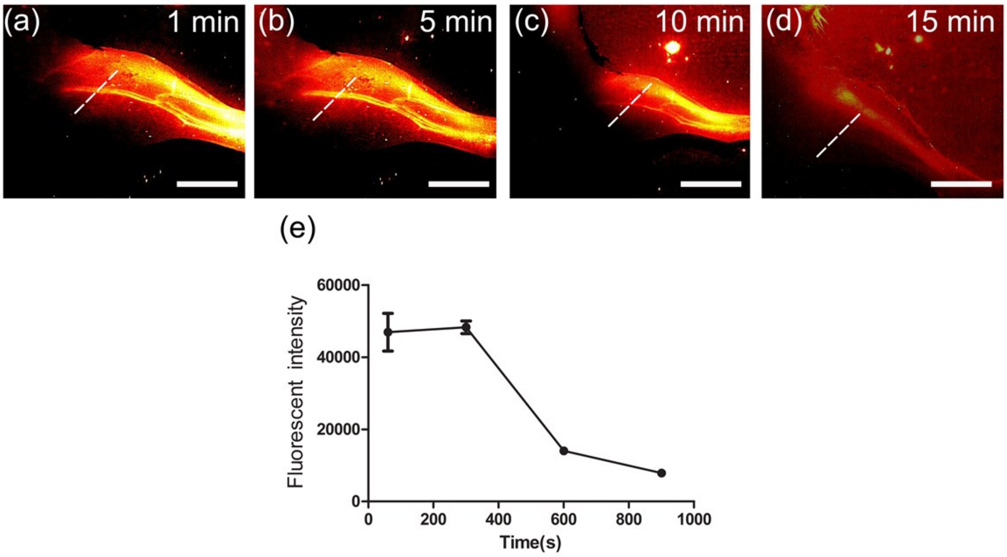

Figure S9. (a-d) Fluorescent imaging exhibited that the hind limb vessel (femoral artery, yellow arrow) were distinguishingly observed under NIR-II imaging and the signal maintained for several minutes. **(e)** A fluorescent intensity analysis representing the intersection point with white-dashed line and the major artery in (a–d), thus half -life of blood circulation ($t_{1/2}$) was calculated as 8.78min. Scale bar: 1.5 cm.

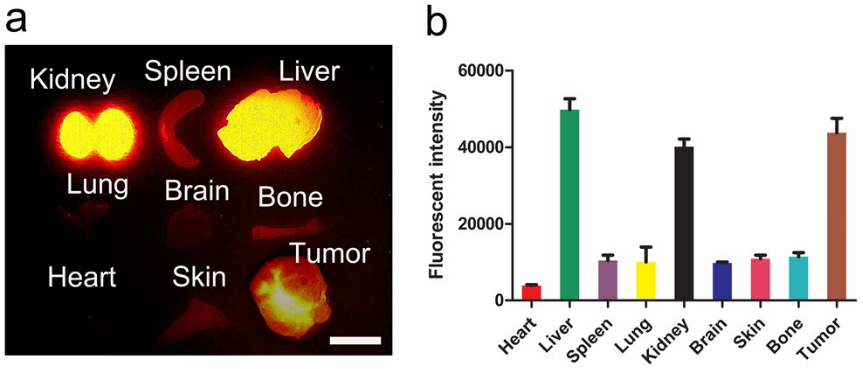

Figure S10. (a) *Ex vivo* fluorescent images of mice bearing with osteosarcoma on day3 demonstrated the favorable contrast using PDFT1032 between tumor and skin. The *ex vivo* fluorescent signal of different organs were shown as **(b)**.Scale bar: 1.5 cm.

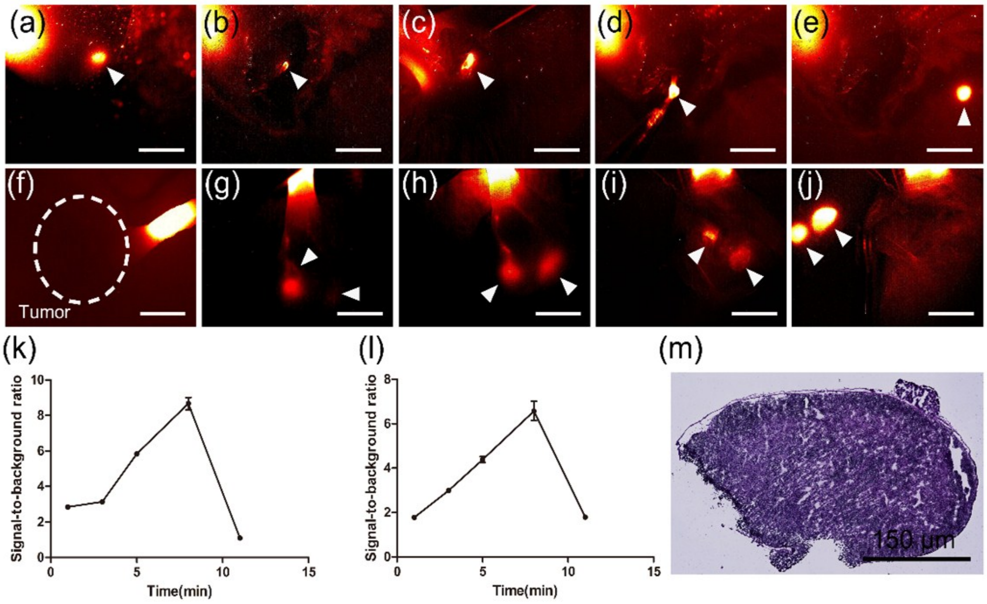

Figure S11. NIR-II image-guided lymph node resection. With the help of NIR-II imaging, the axillary lymph nodes were clearly identified within short time **(a)** and **(b)** and dissected from the ambient tissue **(c)-(e)**, then confirmed by histological analysis **(m)**. **(f)-(j)** Lymph node resection under pathological status. White arrowheads indicate the lymph nodes. During the surgical procedure, the lymph nodes were distinctly identified and distinguished from the ambient tissue because of the favorable signal-to-background ratio (SBR) generated by NIR-II PDFT1032 probe **(k)** and **(l).** Scale bar: 8 mm.

General methods

1. Synthesis of ploymer PDFT

The synthesis method of monomer DF was found from in previously reported.

(1) Synthesis of 3,6-di(furan-2-yl)pyrrolo[3,4-c]pyrrole-1,4(2H,5H)-dione (2). *Tert*-amyl alcohol (50 mL) was heated up to 65 °C under nitrogen atmosphere in a 1000 mL two-neck round-bottom flask. Sodium (4.94 g, 214 mmol) was added into the flask. The solution was stirred at 120 °C. After 12 h, furan-2-carbonitrile (20.0 g, 214 mmol) was poured into the flask. Dimethyl succinate (5.23 g, 35.8 mmol) dropwise added into the flask, and the mixture was stirred for 2 h. Then, cool the reaction mixture to room temperature, and the precipitated was collected and dried under vacuum to obtain the compound *2* (29.4 g, 87% yield) without further purification.

(2) Synthesis of 2,5-dihexadecyl-3,6-di(furan-2-yl)pyrrolo[3,4-*c*]pyrrole -1,4(2*H*,5*H*)-dione (3). Compound 2 (6.90 g, 22.10 mmol) and dry DMF (100 mL) were heated up to 120 °C under N_2 atmosphere. Then, 1-bromohexadecane (16.89 g, 55.26 mmol) was added into the flask. The reaction mixture was heated up to 140 °C and stirred for 3 h. After cooled to room temperature, the reaction mixture was poured into the water, the precipitate was purified by column chromatography. 4.4 g of 3 were isolated. ^{1}H NMR (400 MHz, $CDCl_3$): δ (ppm) = 8.35 (d, J = 3.4 Hz, 2 H), 7.67 (d, J = 1.2 Hz, 2 H), 6.74 (dd, J = 1.7 Hz, 3.7 Hz, 2 H), 4.20-4.09 (m, 4 H), 1.83 – 1.65 (m, 4 H), 1.50 – 1.22 (m, 52 H), 0.94 (t, J = 6.7 Hz, 6 H). ^{13}C NMR (100 MHz, $CDCl_3$): δ (ppm) = 160.9, 145.1, 144.7, 133.7, 120.1, 113.5, 106.5, 42.4, 29.7, 29.6, 29.4, 26.9, 22.7, 14.1. MALDI-TOF MS (m/z): calcd. for $C_{46}H_{72}N_2O_4$ [M^+] = 716.55; found 717.43.

(3) Synthesis of DF. Compound **3** (3.70 g, 5.16 mmol) was dissolved in $CHCl_3$ (100 mL). *N*-

bromosuccinimide (NBS, 0.92 g, 5.16 mmol) was added into the mixture under ice bath. The chloroform was evaporated, and the solid was purified by column chromatography. DF (0.63 g) was isolated (53% yield). ^{1}H NMR (400 MHz, $CDCl_3$): **δ** (ppm) = 8.30 (d, *J* = 3.7 Hz, 2 H), 6.67 (d, *J* = 3.7 Hz, 2 H), 4.13-4.05 (m, 4 H), 1.78 – 1.68 (m, 4 H), 1.52 – 1.19 (m, 52 H), 0.92 (t, *J* = 6.6 Hz, 6 H). ^{13}C NMR (150 MHz, $CDCl_3$, 50 °C): **δ** (ppm) = 160.8, 146.5, 132.8, 126.5, 122.2, 115.7, 106.7, 42.7, 32.1, 30.4, 29.9, 29.8, 29.7, 29.5, 29.4, 27.1, 22.8, 14.2. MALDI-TOF MS (*m/z*): calcd. for $C_{46}H_{70}Br_2N_2O_4$ [M^+] = 874.37; found 874.02.

(4) Synthesis of PDFT polymer. DF (300 mg, 0.342 mmol), T (140.5 mg, 0.342mmol), $Pd_2(dba)_3$ (2 mol %, 6.86 μmol, 6.28 mg), $P(o\text{-}tol)_3$ (8 mol %, 27.44 μmol, 8.34 mg) were mixed in a 20-mL Schlenk tube and then the tube was flushed with N_2 three times. Toluene (10 mL) was added into the tube. After additional 24 h at 110 °C, the mixture was allowed to cool down to room temperature and poured into 100 ml methanol. The methanol solution was then filtered through a Soxhlet thimble. The precipitates were purified via Soxhlet extraction to afford DFT as a dark solid. (287 mg, Mn = 52 kDa, Mw = 80 kDa, PDI = 1.86).

Preparation and characterization of water-soluble PDFT1032

The PDFT polymer (2.5 mg) was dissolved in THF (2.5 mL), and the suspension was filtered through a 20 μm Nylon membrane to afford PDFT as a dark green solution. The dark green solution (0.5ml) was mixed with an aqueous solution of DSPE-mPEG (5 kDa) at a concentration of 1.1 mg/ml with a 1:8 volume ratio for making the PDFT1032. The tetrahydrofuran was removed under N_2. The solution was filtered through a 20 μm Nylon membrane to afford PDFT1032.

2. Experimental Section

2.1 Characterization of PDFT1032

The sizes of the PDFT1032 NIR-II probe were measured by a dynamic light scattering (DLS) instrument (Malvern Instruments Ltd, Southborough, Massachusetts). Ultraviolet-visible (UV-vis)

absorption spectroscopy of the probe was recorded on an Agilent 8453 UV spectrophotometer. Fluorescence was recorded on a Fluoromax-3 spectrophotometer (JobinYvon). The fluorescence emission of phantoms was measured under various filters from 1,000-1,200 nm. Fluorescent stability of PDFT1032 was measured by analysis of mean fluorescent intensity at different time-points after been incubation with phosphate-buffered saline (PBS, Gibco, Life Technologies), fetal bovine serum (FBS, (Gibco, Life Technologies) and Dulbecco's modified Eagle medium (DMEM, Gibco, Life Technologies) at 1,000 nm LP filter with 500 ms exposure time.

2.2 Cell study

B16F10 (mouse melanoma), NIH 3T3 (mouse embryonic fibroblast cell) and 143B (human osteosarcoma) were cultured in Dulbecco's modified Eagle medium (DMEM, Gibco) containing high glucose (Gibco), all of which were supplemented with 10% fetal bovine serum (FBS, Gibco) and 1% penicillin–streptomycin. The cells were expanded in tissue culture dishes and kept in a humidified atmosphere of 5% CO_2 at 37 °C. The medium was changed every other day. A confluent monolayer was detached with 0.5% trypsin, and dissociated into a single-cell suspension for inoculation.

The potential cytotoxicity of PDFT1032 on NIH3T3 cells was examined by employing the MTT (3-(4,5-dimethylthiazol-2-yl)-2,5-diphenyl tetrazolium bromide) (Sigma-Aldrich, St. Louis, USA) assay. The cells with a density of 5×10^3 per well were seeded in 96-well plates. After 24 h, the medium of 96-well plates was replaced with 100 μL of medium containing different concentration of PDFT1032 and incubated followed by 48 h. After that, MTT (10 μL,0.5 mg/mL) solution was added to each well and incubated for 4 h at 37 °C. The supernatant was removed and the residues were lysed with 200 μL of dimethyl sulfoxide (DMSO). The absorbance value was

recorded at 490 nm using a micro plate reader. The absorbance of the untreated cells was used as a control and the reference value for calculating 100% cellular viability. The half-maximal inhibitory concentration (IC_{50}) was measured via the MTT assay.

2.3 Animal studies

C57BL/6 and Nude mice (Charles River Laboratories, USA) were maintained in pathogen free conditions until imaging.

For osteosarcoma inoculation, four to six-week-old nude mice (n = 3) were inoculated with five million 143B cells in 100 μL of serum-free medium at the right front shoulder for subcutaneous model, or one million 143B cells in 30 μL of serum-free medium into the marrow cavity of left tibia for orthotopic model (n = 3), respectively. Tumors were allowed to grow for approximately 10-21 days before imaging. For assessing the vascular embolization therapy, four to six-week-old nude mice (n = 3) were inoculated with five million 143B cells in 100 μL of serum-free medium at the proximal region of right thigh. Tumors were allowed to grow for approximately 7-10 days before imaging. For melanoma inoculation, four to six-week-old nude mice were inoculated subcutaneously with five million B16F10 cells in 100 μL of serum-free medium at the right shoulder. Tumors were allowed to grow for 10-14 days.

2.4 NIR-II image-guided surgery

Surgery was performed by a skilled surgeon according to the NIR-II imaging screening results at 1,000 nm LP filter with 200 ms exposure time. The tumor tissue of osteosarcoma were resected and embedded by *optimal cutting temperature* (*OCT)* compound (Tissue-Tek®, Sakura Finetek, USA), and then cut into 6 μm sections in the cryostat at -20 ^{o}C with a microtome and transferred onto microscope slides for hematoxylin and eosin (H&E) using Shandon^{TM} rapid

Chrome kit (Thermo Scientific, USA). During the whole process of imaging, the mice was kept anaesthetized by a nose cone delivering 2 L/min O_2 gas mixed with 3% isoflurane.

2.5 NIR-II imaging setup

All NIR-II images including pre- and post- injection of the probe were collected on a 320×256 pixel two-dimensional InGaAs array (Princeton Instruments). The excitation laser was an 808 nm laser diode at a power density of 0.3 W cm^{-2}. Emission was typically collected with a 1,000 nm LP (long-pass) filter. A lens set was used to obtain tunable magnifications ranging from 1× (whole body) to 2.5× (high magnification) by changing the relative position of two NIR achromats (75 mm and 200 mm, Thorlabs). A binning of one and a variable exposure time were used for the InGaAs camera (320 × 256 pixel) to capture images in the NIR-II window. Detailed imaging procedures to visualize regions of interest in mice were described in the sections below. Image J software were used for analyzing the images. The tumor-background ratio was measured by selecting the surrounding normal tissue as background.

3 Statistical analysis

The fluorescence measurement was performed to quantitate NIR fluorescence signal intensity through the Image J 1.45x software (National Institutes of Health, Bethesda, MD). Data are given as mean ± SD (standard deviation).
